# Research on the visual search behavior and decision-making ability of basketball referees

**DOI:** 10.3389/fpsyg.2025.1682389

**Published:** 2025-10-13

**Authors:** Rishu Wang, Long Chen, Yidong Wu, Qi Zhang

**Affiliations:** ^1^School of Athletic Performance, Shanghai University of Sport, Shanghai, China; ^2^School of Physical Education and Equestrian, Wuhan Business University, Wuhan, China; ^3^School of Economics and Management, Shanghai University of Sport, Shanghai, China

**Keywords:** basketball referee, visual search behavior, decision-making, experience, referee

## Abstract

**Background:**

In sports, basketball referees have to face high pressure and time constraints, efficient visual search behavior and decision-making particularly important.

**Methods:**

This study compared different levels of experience (expert group, *n* = 10; non-expert groups, *n* = 10) examined the visual search behavior and decision-making ability of basketball referees when watching 20 game video clips through eye movement technology.

**Results:**

The results showed that, compared with the non-expert group, the expert group had higher decision-making accuracy (*p* < 0.01), and the percentage of fixations time was longer in the central area (*p* < 0.01), the outer area (*p* < 0.01) and the percentage of fixations time in the invalid area (*p* < 0.05). However, there were no significant differences in the number of fixations (*p* = 0.904), fixations duration (*p* = 0.363) and entropy (*p* = 0.213) between the expert group and non-experts.

**Conclusion:**

Our research indicates that there are significant differences in the visual search behaviors of basketball referees with different experiences. These data can provide valuable insights into the visual search patterns of basketball referees in real game environments and emphasize the importance of refereeing expertise for basketball referees.

## 1 Introduction

Referees are one of the most important components of sports competitions. Only those with special physical and mental abilities can make correct decisions (Bar-Eli et al., 2011). In fast-paced team games (such as basketball and football), the role of these abilities is even more important. Basketball referees need to have high requirements for interaction and physical movement, and often need to handle a large number of cues (MacMahon et al., 2014). Because in the basketball game, referees not only need to observe players and teammates simultaneously, but also make decisions under high pressure and time constraints (Klatt et al., 2021). Referees’ decisions are based on the performance of their personal perceptual cognitive skills. For example, football referees make an average of 162 decisions per game (Helsen and Bultynck, 2004). However, a key and interesting question is: How do basketball referees make these decisions in fast-paced and complex situations.

Whether it is time constraints, complex competition scenarios or potential pressure conditions, referees need to make accurate and fair decisions (Samuel, 2015). The sports officials’ decision-making model explains how individuals interpret dynamic environments through social knowledge and cognitive processes. It emphasizes that perception and mental representation are the processes before decision-making and decision execution (Kostrna and Tenenbaum, 2021). Mental representations control the perceptual and cognitive processes, including emotions, information processing, attention, expectations, information integration and performance. Decision-making is mainly influenced by mental representations (Kostrna and Tenenbaum, 2021). Decision making involves choosing a series of actions from among a certain class of alternatives with a specific goal in mind. According to this theory, the aim of decision making is to increase the achievements or raise the level of expectations and to use the information in order to achieve this goal (Tenenbaum et al., 2007; Gorman et al., 2015). It is clear that experts have cognitive-perceptual skills such as pattern recognition and recall based on the perception of basic visual information that helps make the right decision (Van Maarseveen et al., 2015; Klostermann and Moeinirad, 2020). How referees in complicated situations with a time limit to make a decision, one possible way is through the visual search behavior to gain knowledge support to make a decision.

Perceptive skills allow to efficiently manage and attend to highly relevant information that, according to previously acquired knowledge, facilitates the selection of appropriate make decisions under time pressure (Marteniuk, 1976). Existing research believes that efficient visual gaze behavior is an important factor in decision-making performance in complex movements (Williams and Ward, 2007). In the competition situation, referees mainly make decisions by visual searching for important stimulus information. Visual search can improve decision performance by extracting environmental information (Vater et al., 2019), and visual search behavior represents how the referee’s visual system detects relevant information (Henderson, 2003).

Previous research has shown that experts’ visual search strategies are less number of fixations and fixations duration (Mann et al., 2007). Today, many studies have reported that visual search strategies perform differently in different sports (North et al., 2009; Roca et al., 2011; Ziv, 2020). A lot of research supports this view. Expert athletes spend more number of fixations and fixations duration in the main areas of interest that can provide task-related information (Brams et al., 2019). In terms of referees, there are also reports of similar results. This finding is consistent with the information-reduction hypothesis (Haider and Frensch, 1999), which argues that through experience, experts selectively allocate attention to the task-relevant areas of the and ignoring task-redundant areas. The referee’s expertise cannot evaluate visual search strategies based on the number of fixations and fixations duration, but should focus more on the gaze behavior of the area. The perceived cognitive needs of referees in different sports events are different, mainly due to the unique characteristics of sports events. For example, basketball referees are interactors, and their characteristics usually require handle a large number of game clues (MacMahon et al., 2014), and reasonable allocation of attention between the referee’s responsibility area, ball, players and companions in the game scenario (Klatt et al., 2021). Recent research has found that when interactors (such as hockey and soccer referees) make decisions, referees of different expertise do not differ in the number of fixations and fixations duration (Rafiee et al., 2015). But experienced referees will spend more time focusing on the body parts of the offensive players (Ruiz et al., 2023).

The visual search behavior of referees relies on task characteristics (Kredel et al., 2017), in which experts tend to use shorter fixations duration in dynamic tasks, while in relatively static tasks, longer fixations duration (Gegenfurtner et al., 2011). The latest research shows that by introducing AI-based eye movement analysis for basketball games, it is more helpful to understand their visual attention patterns, objects of focus, duration, and physiological responses, etc. It can be seen from this that by leveraging AI technology and wearable devices, a deeper understanding of the cognitive and decision-making processes adopted by referees in high-pressure sports environments can be achieved (Lozzi et al., 2025). In basketball, predecessors compared the visual search behavior of basketball referees of different levels of experience. Although no differences in the number of fixations and fixations duration between experts and novices, some studies suggested that the referee’s gaze behavior varies depending on their position on the field and their level of experience. Experienced referees demonstrate efficient gaze patterns and can focus on key areas more quickly and accurately. However, referees who are far from the ball often overlook the behavior in the no-ball area, indicating that it is necessary to improve the distribution of visual attention (Alemanno et al., 2025). Knowing when to see where, extracting tasks-related information and ignoring irrelevant information is crucial for efficient decision-making. Therefore, this study will further explore the differences in visual search behaviors between expert and non-expert referees by dividing areas of interest (e.g., trunk, legs, feet, arms, etc.).

In conclusion, our aim is to analyze the visual search behavior and decision-making ability of referees in real basketball game scenarios, and we attempt to determine a visual search pattern for basketball referees that distinguishes experts from non-experts. For this purpose, we obtain video clips from the real perspective of the referee (the lead referee location) to restore the real game situation as much as possible. We particularly emphasize the analysis of the referees’ gaze positions and establish three areas of interest to compare the differences in gaze behaviors between expert and non-expert referees. In this study, we predict that the number of fixations and the fixations duration by the expert group basketball referees may be similar to the non-expert group, but experts will pay more attention to task-related and information-rich key positions, which is in line with the information reduction hypothesis. This visual search strategy is particularly prominent among interactors (such as basketball referees and football referees), as they need to observe more visual cues than reactors and monitors. Furthermore, we predict that the decision-making accuracy of the expert group will be higher than the non-expert group, which may be related to their expectation ability and long-term working memory ability.

## 2 Materials and methods

### 2.1 Participants

This study was analyzed using G*Power 3.1.9.2 software with an effect size of 0.4, an *α* level of 0.05 and an power of 0.08. The results indicated that 52 subjects were required. However, due to the particularity of the research subjects (such as the total number of professional league referees in the Shanghai area is relatively small, and the limitations of time and funds), it is difficult to reach the sample size calculated theoretically in the actual study. We eventually recruited 20 subjects. Furthermore, based on previous studies on referee visual search and decision-making, we find that the sample size selection is similar to ours, for example (Ruiz et al., 2023, 2024) recruited 16 basketball referees, and the study (Klatt et al., 2021) recruited 9 basketball referees. The relatively larger number of participants was approximately 30 (Hancock and Ste-Marie, 2013; Moore et al., 2019). These studies show that although the sample size is small, some valuable insights and conclusions can be drawn. To ensure the data quality of all subjects, we adopted strict data inclusion and exclusion criteria as well as quality control. Ensure that all the data of the recruited candidates are included in the data analysis, and there are no cases excluded due to poor quality.

All the participants were divided into two groups. The referees who officiate in the country’s top-level basketball league are classified as expert groups (*N* = 10, *M*_age_ ± *SD* = 36.9 ± 7.49 years, *M*_experience_ ± *SD* = 15.1 ± 3.98 years). Many of whom were refereeing, or had refereed, at international level basketball games. Referees officiating in various provinces, cities and regions were classified as non-expert groups (*N* = 10, *M*_age_ ± *SD* = 22.3 ± 2.91 years, *M*_experience_ ± *SD* = 5.3 ± 1.34 years). Who were from University who refereed at lower competitive levels, but had little experience refereeing professionally. All the Participants’ vision reached 5.0 or the corrected level of 5.0, and they all signed the written informed consent form before the experiment. This study was approved by the Ethics Committee of Shanghai University of Sport (Approval No.: 102772024RT070).

### 2.2 Apparatus

Participants watched the video clips at a distance of approximately 42–62 cm from the computer. The video clips were presented on a 23.8-inch computer display screen (EIZO EV2451, with a resolution of 1920 × 1080 and a viewing Angle of 178/178 degrees). The video had no sound to avoid the influence of crowd noise. During the experiment, the Eyelink Portable Duo eye tracker (SMI, Boston, MA), with dimensions of 21 cm x 4.5 cm x 11 cm, weighing approximately 1 kg, having a binocular sampling rate as high as 2,000 hz and an accuracy of 0.5 degrees, located beneath the display screen the eye tracker, which is connected to a laptop (Lenovo, ThinkPad) via a USB cable, will record the visual behaviors of the subjects. In this study, an eye tracker placed on a desktop was used. To complete the sampling more conveniently and efficiently, a stand was installed beside the table where the screen was placed. The position of the stand can be adjusted. This is because the subjects have different heights, and the best position to watch the video needs to be selected by adjusting the position of the stand.

### 2.3 Video clips

**Figure 1:**
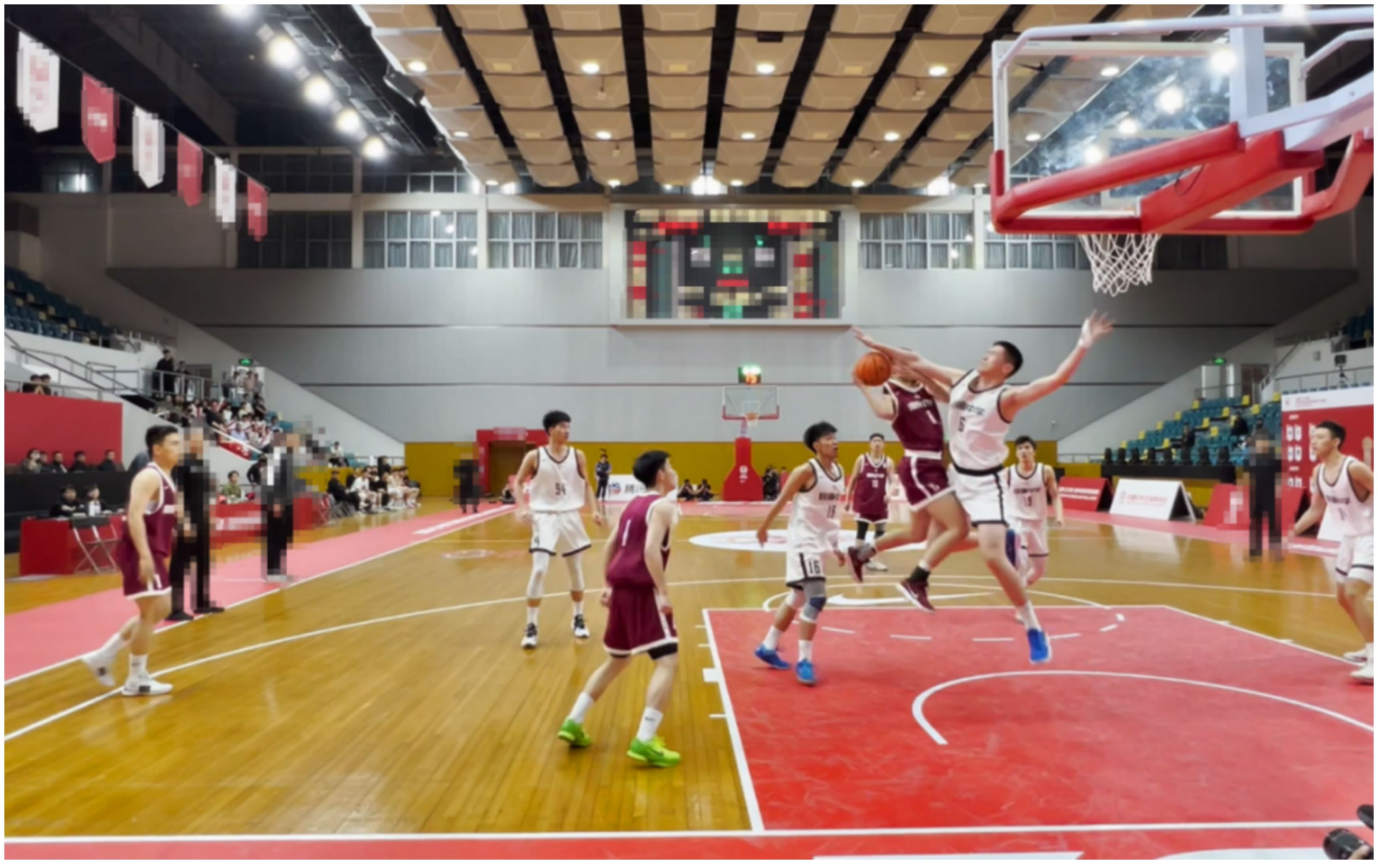
Examples of experimental materials.

To design the experimental video clips, we carried out several steps. First of all, all videos are from the game clips of the China Nike High School Basketball League (the highest-level high school basketball competition in China) shot from the perspective of the lead referee (see Figure 1). The inclusion criteria for the videos are: ① there is physical contact; ② in an offensive or defensive situation; ③ violation situation occurs in the area of the lead referee; ④ there is only one foul or one violation in the video. After the author’s initial screening, 30 video clips were saved. However, this cannot be directly used as experimental material and needs to be further reviewed by experts. Secondly, the selection process of the experimental video clips was rather difficult. Therefore, a video review team (including the author, referee supervisors, referee lecturers and technical representatives from the Chinese Basketball Association) was established in this study, mainly to review whether the edited videos could be used as experimental materials. The team reaches a consensus on the penalty decision (for example, whether to commit a foul, and if so, what is the behavior of the foul?). And rule out particularly obvious fouls or violations. After review by the group, 10 videos were saved. Thirdly, we must meticulously edit these 10 videos. The videos start when ball-handling offense and defense situations occur (for example, the blue team fails to score while the red team gets the rebound and holds the ball until a defender appears in front of them), and end before the referee makes a decision. The referees in the video were also covered to prevent them from attracting the attention and interest of the subjects when they were watching the video. Finally, the referee supervisor, referee lecturers and technical representatives reviewed these 10 videos and checked the standards such as video length and clarity. Therefore, 10 video clips of the competition scenes with durations ranging from 10 to 30 s were generated. Each video contains only one possible violation. The video is played silently, eliminating crowds, referees, viewers, scores and advertisements to reduce the number of distractions.

### 2.4 Variables

#### 2.4.1 Decision-making accuracy

After the two competition supervisors of the review panel watched each video, their decisions on the video were regarded as correct ones. Decision-making accuracy was calculated as the total number of decisions (displayed as a percentage) that were in correspondence with the reference decision (as Spitz et al., 2016).

#### 2.4.2 Gaze behavior

**Figure 2:**
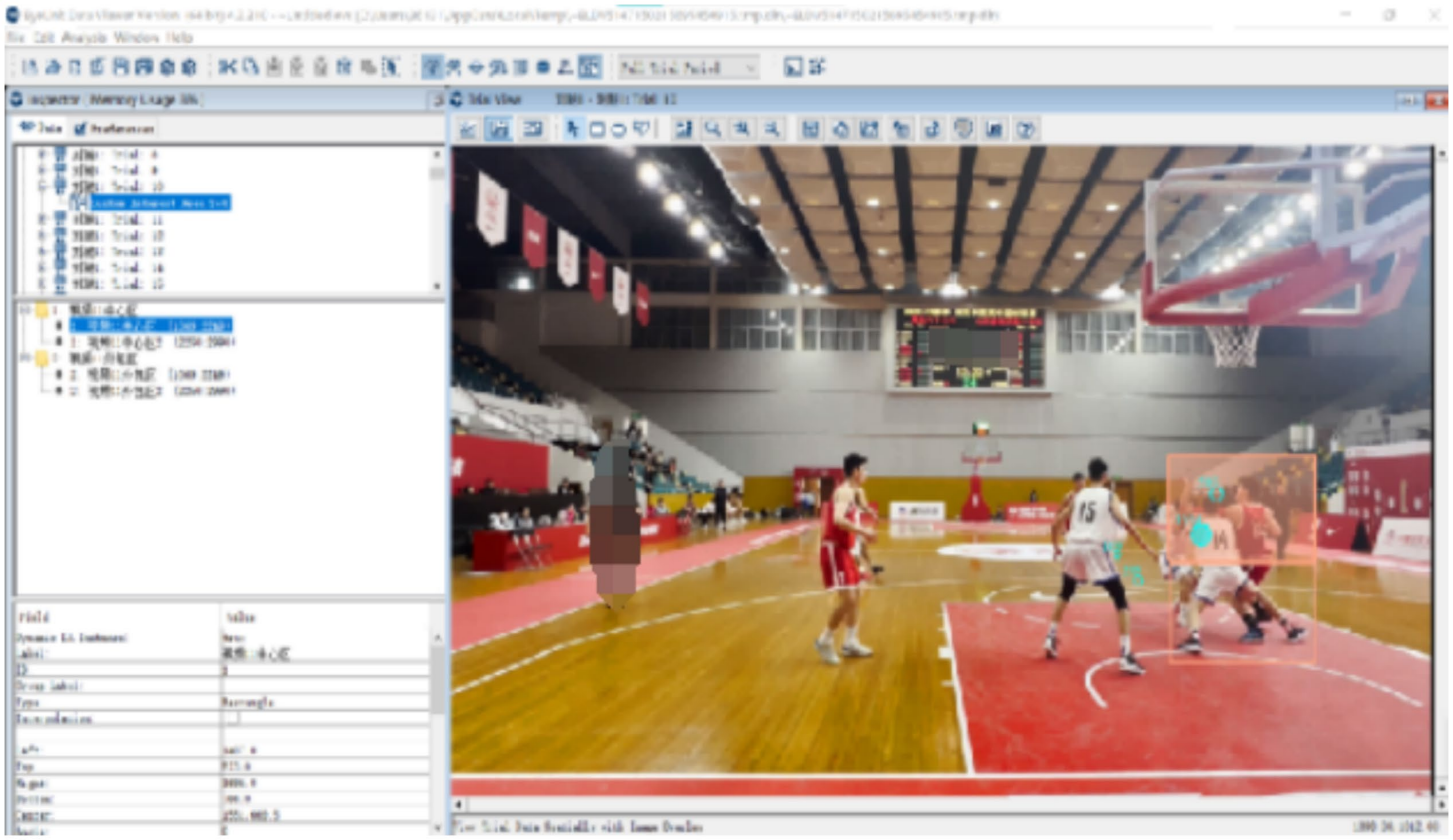
The orange area represents the central area, the white area represents the outer area, and the rest are invalid area.

A fixation was defined as a gaze that was maintained on a location within 1° of visual angle for a minimum of 120 ms (Vickers, 2007). Four gaze measures were assessed for each of the 10 video clips. ① Number of fixations, ② fixations duration, ③ percentage of fixations time for different locations, ④ entropy, ⑤ fixation heat map. The number of fixations and fixations duration reflect the need for information processing and the allocation of attention. Firstly, the number of fixations referred to the number of times a participant moved their eyes and fixated on a point for a minimum of 100 ms (Hancock and Ste-Marie, 2013). Secondly, fixations duration was the length of time participants focused on an area before fixating elsewhere (Hancock and Ste-Marie, 2013). Thirdly, Percentage of fixations time referred to the percentage of total fixations time spent fixating each location (as the central area, the outer area, the invalid area) (Roca et al., 2011). The central area refers to the area where key information can be obtained by looking at this area. The outer area refers to the area where a small amount of information related to decision-making is obtained by looking at this area, and the invalid area refers to no information related to decision-making (Moore et al., 2019). The division of the area of interest is achieved through interviews with referee supervisors. The central area includes the torso and arms of the ball handler, the torso and arms of the defender, and the contact points. The outer area includes the lower limbs of the ball handler and the defender, as well as the torso, arms and lower limbs of the assist defender. The invalid area contains the bodies of the remaining players (see Figure 2). Using 10 selected video clips as materials, the area of interest for each video is drawn on the Data Viewer software. Fourth, entropy refers to the uncertainty within a system, indicating the variability of gaze behavior. While different measures of entropy exist (Allsop and Gray, 2014), Shannon entropy derives from information theory (Shannon, 1948), and expresses the information contained within a probability distribution in “bits.” Entropy was calculated as the sum of the logarithm of all probabilities in the given state space, $H(x)=-\sum_{i=1}^{n}P(x_{i})\log_{b}P(x_{i})$ (Shannon, 1948). In short, lower entropy values therefore reflected gaze behavior that was focused on particular fixation locations, rather than distributed or spread evenly across all locations. Finally, fixation heat map uses the warmth or coolness of colors (such as green-yellow-red) to represent the spatial distribution density of fixation points. The hotter the color (such as red), the longer the total duration of fixation or the more times the area is gazed at by all participants.

### 2.5 Procedure

**Figure 3:**
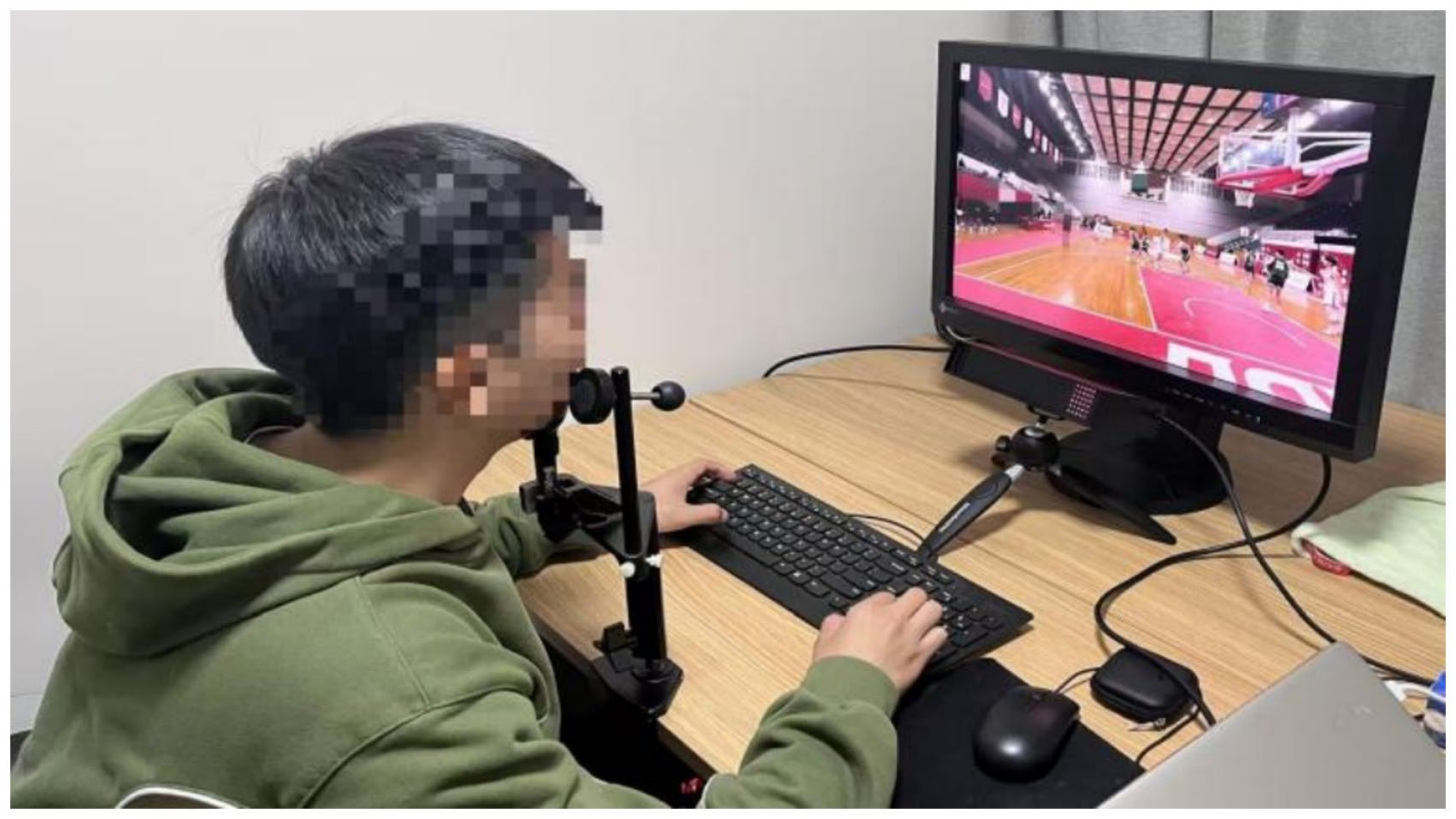
Experimental process.

**Figure 4:**
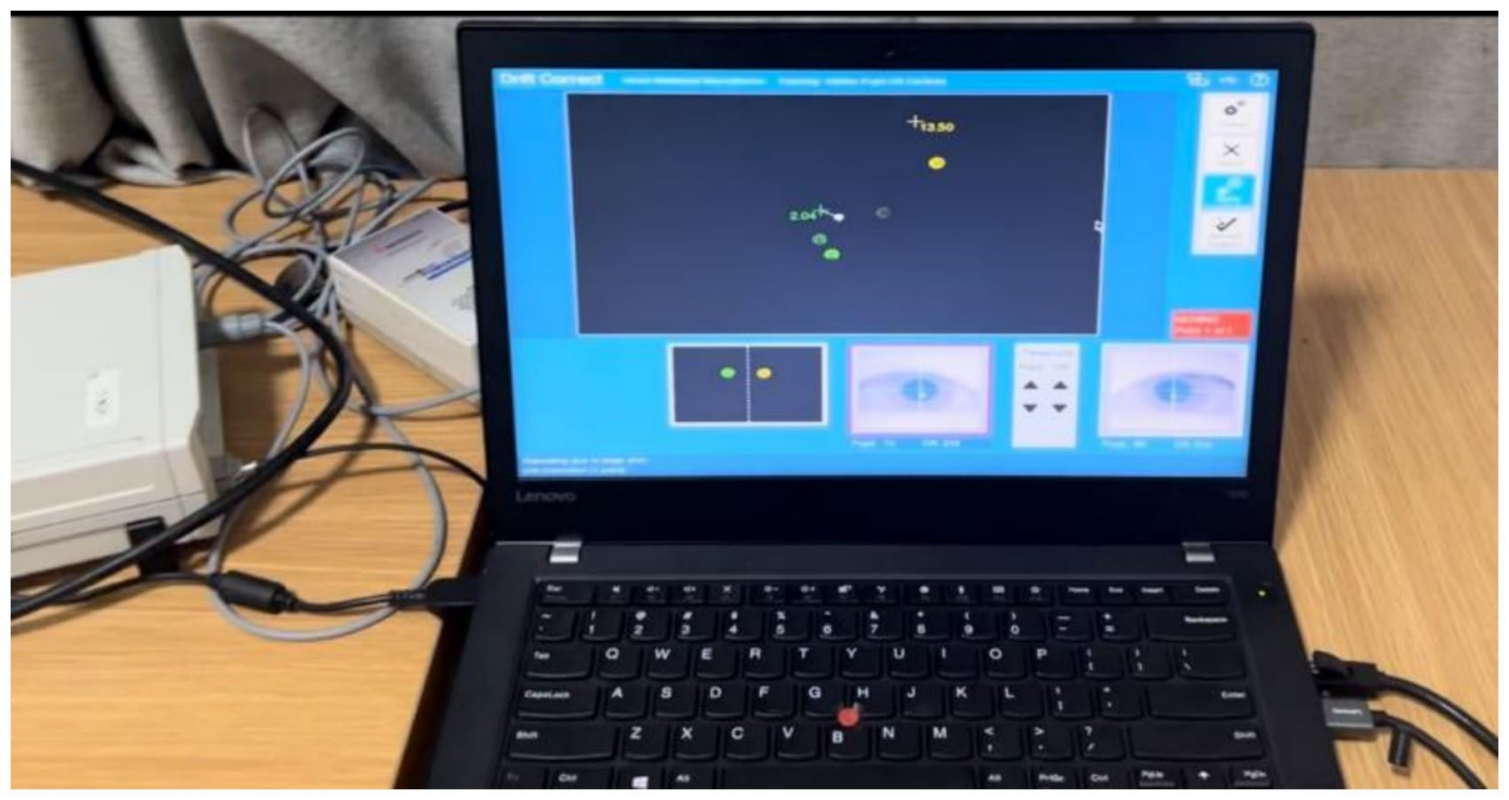
The recording process of the eye tracker.

Participants first fill out the personal information form and provide a written informed consent form. Next, the participants adjusted the scaffolds and performed 9-point grid calibration. Then, watch a practice video to familiarize yourself with the experimental process. We provide the participants with standardized and detailed oral explanations of the tasks. Participants are required to watch each video clip and then select the answer as soon as possible after the options are displayed on the screen. For each segment, participants have to choose one of the five decisions: ① travel, ② no call (no violation occurred), ③ offensive foul, ④ defensive foul, ⑤ cheating foul, ⑥ traveling. After getting familiar with the experimental process, ask the participants whether they fully understand the task. Subsequently, the participants watched 10 videos and made decisions, while their gaze behaviors and decisions were recorded. To ensure the accuracy of the data, eye movement calibration was performed on the subjects before each video was played. None of the videos were replayed, and no feedback was given to the participants between the clips. Finally, the eye tracker was removed and the situation of the participants was reported and thanked (see Figures 3, 4).

### 2.6 Statistical analysis

In this study, the group (expert group and non-expert group) was taken as the independent variable, and the number of fixations, fixations duration, Percentage of fixations time for different locations, the entropy, and the decision-making accuracy were taken as the dependent variables. Firstly, the normality of the data was examined. Each dependent variable conformed to a normal distribution. Meanwhile, all the tests conformed to the homogeneity of variance hypothesis (Levene test, *p* > 0.05; Field, 2013). Secondly, one-way ANOVAS analysis of variance and correlation analysis were used. Explore the differences of each dependent variable between groups and analyze the various data among different groups. *p*-value less than 0.05 is considered statistically significant. All the analyses were conducted using IBM SPSS 26.

Data inclusion criteria: All participants ① complete all experimental tasks in full; ② the eye tracker calibration was successful. ③ The overall eye-movement data tracking loss rate is below the threshold of 5%. We strictly control the quality control standards of the experiments. For instance, we conduct strict calibration before each experiment, monitor the data quality in real time during the experiments, and manually and automatically check the videos and data. All the data of the subjects participating in the experiment met the quality requirements, and no participant was excluded due to poor data quality. Ensured the completeness and robustness of the research.

## 3 Results

The purpose of this study was to explore the differences in visual search behavior and decision-making accuracy between experts and non-experts in basketball referees.

### 3.1 Number of fixations

**Figure 5:**
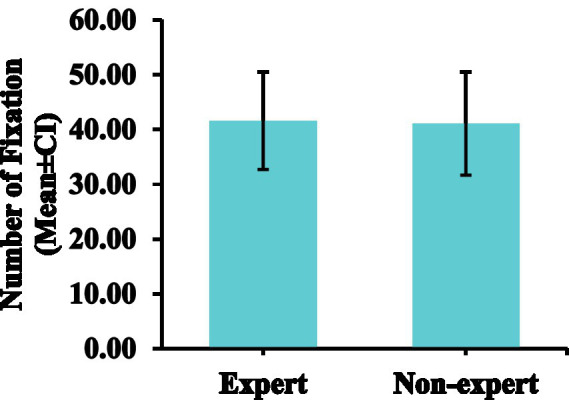
Comparison of the number of fixations between expert and non-expert referees. Error bars are ±1 SEM.

The number of fixations of non-expert referees (*M* = 41.1, *SD* = 9.45) was similar to that of expert referees (*M* = 41.6, *SD* = 8.89). There was no significant difference in the number of fixations between expert and non-expert referees [*F*(1, 18) = 0.015, *p* = 0.904, ƞp^2^ = 0.001]. The number of fixations data are presented in Tables 1, 2 and Figure 5.

**Table 1:** Mean (standard deviation) number of fixations, fixations duration, percentage of fixations time in the central area, percentage of fixations time in the outer area, percentage of fixations time in the invalid area, entropy, decision-making accuracy.

Measures	Group	
	Expert	Non-Expert
Number of fixations	41.60 (8.89)	41.10 (9.45)
Fixations duration (s)	18.19 (1.98)	19.33 (3.30)
Percentage of fixations time in the central area (%)	80.38 (4.55)	53.01 (7.41)
Percentage of fixations time in the outer area (%)	12.90 (3.54)	34.49 (5.02)
Percentage of fixations time in the invalid area (%)	6.71 (4.21)	12.81 (6.82)
Entropy (bits)	0.50 (0.18)	0.60 (0.20)
Decision-making accuracy (%)	78.00 (7.89)	46.00 (8.43)

**Table 2:** The analysis of variance results of number of fixations, fixations duration, percentage of fixations time in the central area, percentage of fixations time in the outer area, percentage of fixations time in the invalid area, entropy, decision-making accuracy.

Measures	*F*	*P*	ƞp^2^
Number of fixations	*F*(1, 18) = 0.015	0.904	0.001
Fixations duration	*F*(1, 18) = 0.871	0.363	0.046
Percentage of fixations time in the central area	*F*(1, 18) = 99.131	<0.01	0.846
Percentage of fixations time in the outer area	*F*(1, 18) = 123.485	<0.01	0.873
Percentage of fixations time in the invalid area	*F*(1, 18) = 5.796	<0.05	0.244
Entropy	*F*(1, 18) = 1.645	0.213	0.058
Decision-making accuracy	*F*(1, 18) = 0.768	<0.01	0.810

### 3.2 Fixations duration

**Figure 6:**
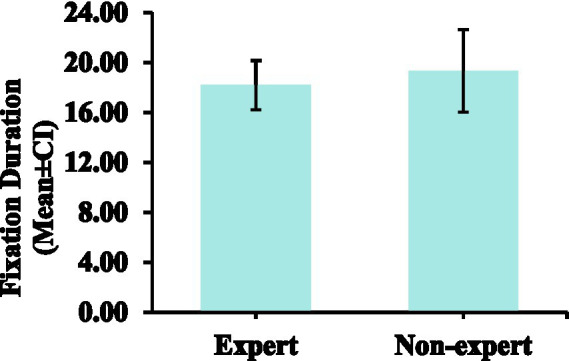
Comparison of the fixations duration between expert and non-expert referees. Error bars are ±1 SEM.

The fixations duration of non-expert referees (*M* = 19.33, *SD* = 3.30) was similar to that of expert referees (*M* = 18.19, *SD* = 1.98). There was no significant difference in fixations duration between expert and non-expert referees [*F*(1, 18) = 0.871, *p* = 0.363, ƞp^2^ = 0.046]. The fixations duration data are presented in Tables 1, 2 and Figure 6.

### 3.3 Percentage of fixations time for different locations

#### 3.3.1 Percentage of fixations time in the central area

**Figure 7:**
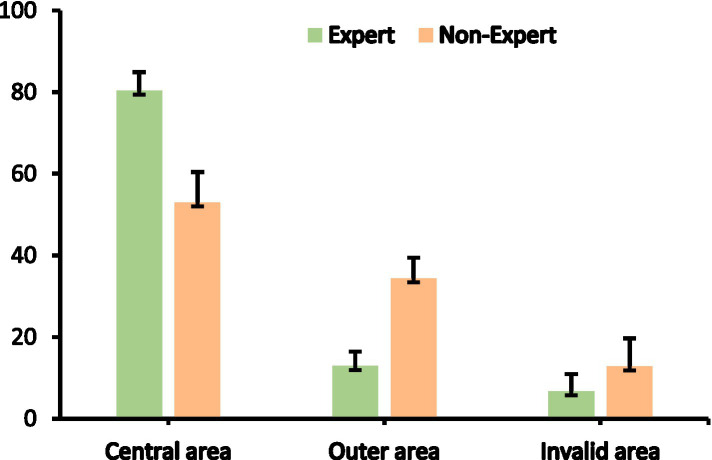
Percentage of fixations time in the central area, outer area and invalid area between expert and non-expert referees. Error bars are ±1 SEM.

The percentage of fixations time of expert referees in the central area (*M* = 80.38, *SD* = 4.55) was significantly higher than that of non-expert referees (*M* = 53.01, *SD* = 7.41). There was a significant difference in the percentage of fixations time between expert and non-expert referees in the central area [*F*(1, 18) = 99.131, *p* < 0.01, ƞp^2^ = 0.846]. The percentage of fixations time in the central area data are presented in Tables 1, 2 and Figure 7.

#### 3.3.2 Percentage of fixations time in the outer area

The percentage of fixations time of expert referees in the outer area (*M* = 12.90, *SD* = 3.54) was significantly lower than that of non-expert referees (*M* = 34.49, *SD* = 5.02). There was a significant difference in the percentage of fixations time between expert and non-expert referees in the outer area [*F*(1, 18) = 123.485, *p* < 0.01, ƞp^2^ = 0.873]. The percentage of fixations time in the outer area data are presented in Tables 1, 2 and Figure 7.

#### 3.3.3 Percentage of fixations time in the invalid area

The percentage of fixations time of expert referees in the invalid area (*M* = 6.71, *SD* = 4.21) was similar to that of non-expert referees (*M* = 12.81, *SD* = 6.82). There was no significant difference in the percentage of fixations time in the invalid area between expert and non-expert referees [*F*(1, 18) = 5.796, *p* < 0.05, ƞp^2^ = 0.244]. The percentage of fixations time in the invalid area data are presented in Tables 1, 2 and Figure 7.

### 3.4 Entropy

**Figure 8:**
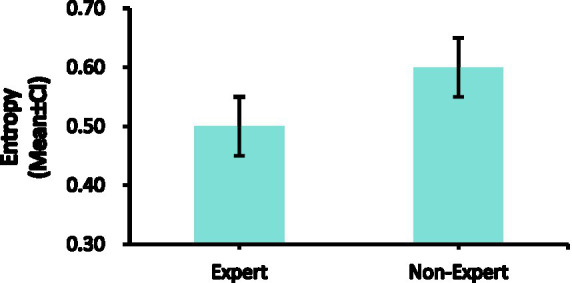
Comparison of the entropy between expert and non-expert referees. Error bars are ±1 SEM.

The entropy of expert referees (*M* = 0.50, *SD* = 0.18) is similar to that of non-expert referees (*M* = 0.60, *SD* = 0.20). There was no significant difference in entropy between expert and non-expert referees [*F*(1, 18) = 1.645, *p* > 0.213, ƞp^2^ = 0.058]. The entropy data are presented in Tables 1, 2 and Figure 8.

### 3.5 Fixation heat map

There are certain differences in the gaze distribution between expert referees and non-expert referees. Expert referees have paid a great deal of attention to the central area, but very little to the outer area and the invalid area. And, the experts focus was mainly on the arms and trunks of the defenders and offensive players. However, non-expert referees focus on relatively scattered areas and cannot obtain the most crucial information in a short period of time.

### 3.6 Decision-making accuracy

**Figure 9:**
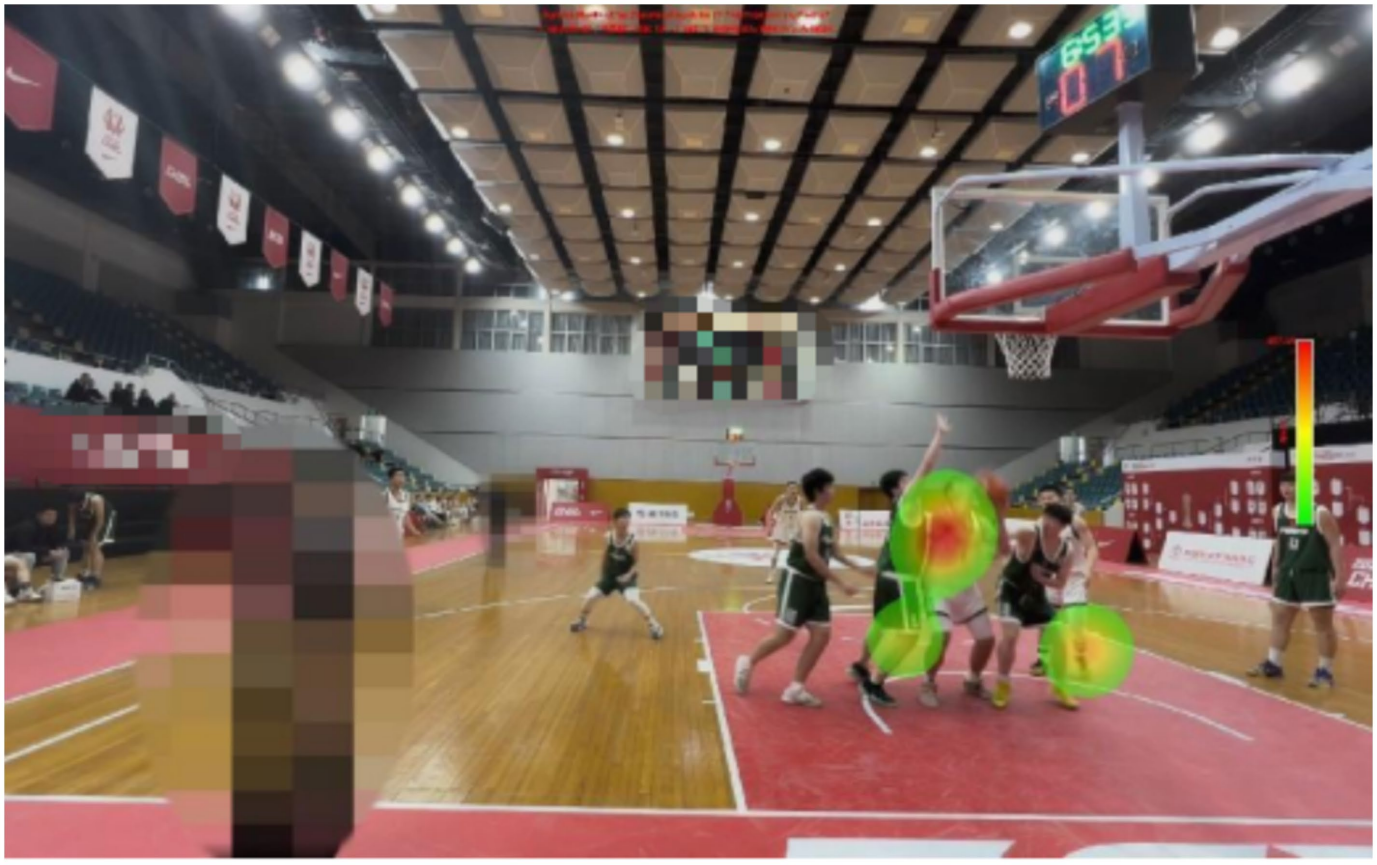
Heat map of a representative non-expert referee.

**Figure 10:**
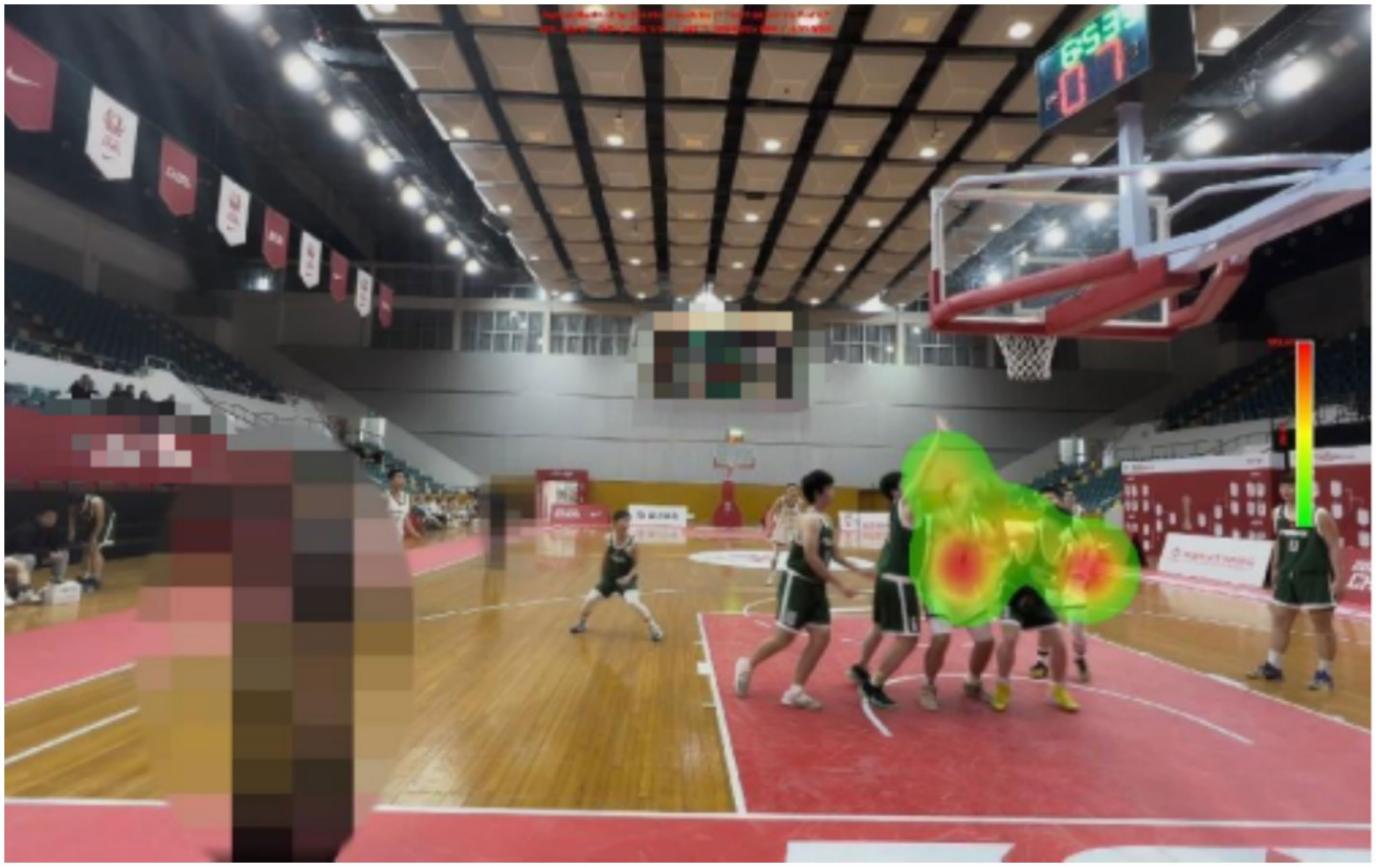
Heat map of a representative expert referee.

**Figure 11:**
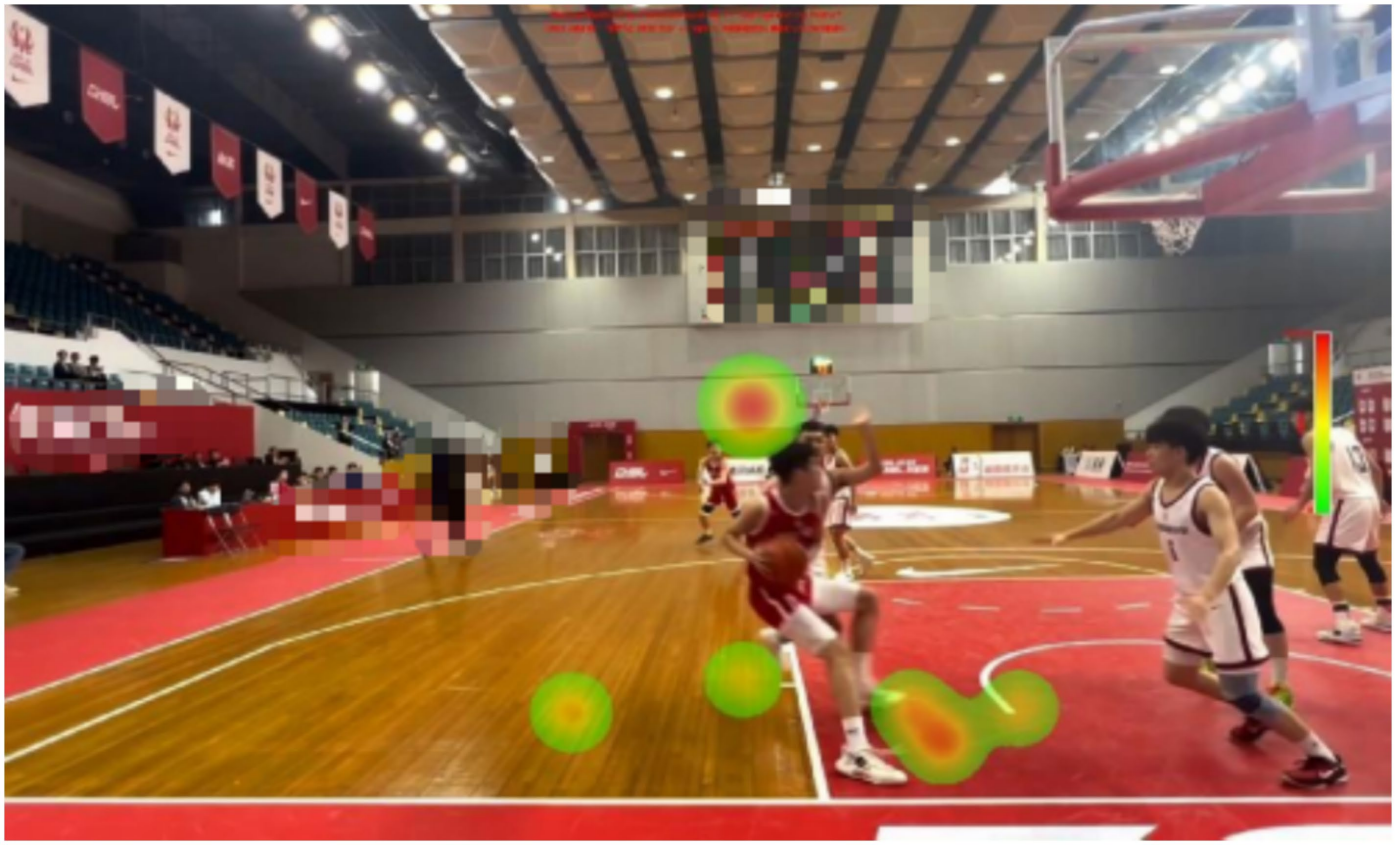
Heat map of a representative non-expert referee.

**Figure 12:**
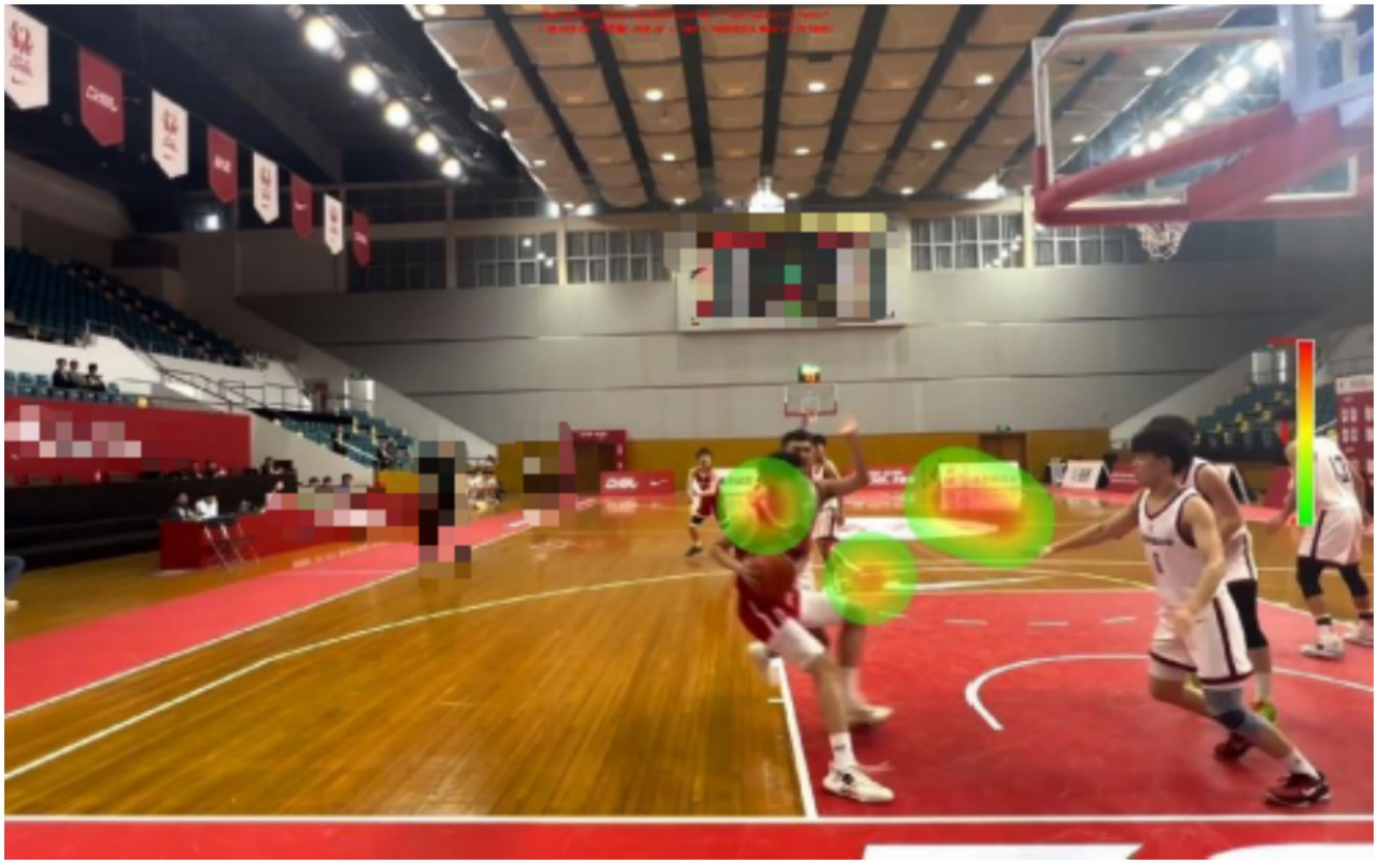
Heat map of a representative expert referee.

**Figure 13:**
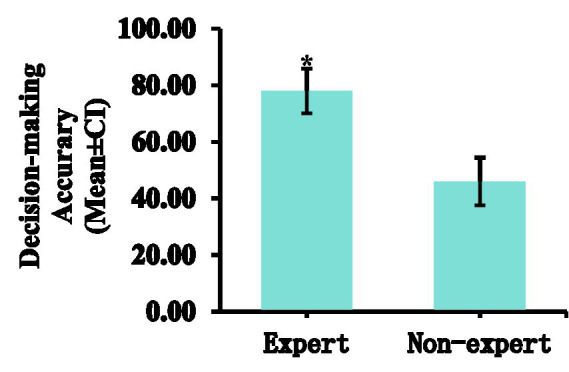
Comparison of the decision-making accuracy between expert and non-expert referees. Error bars are ±1 SEM.

The expert referees were significantly higher than the non-expert referees (*M* = 46.00, *SD* = 7.89) in decision-making accuracy. There was a significant difference in the decision-making accuracy between expert and non-expert referees [*F*(1, 18) = 76.800, *p* < 0.01, ƞp^2^ = 0.810]. The percentage of fixations time in the invalid area data are presented in Tables 1, 2 and Figures 9–13.

## 4 Discussion

This study employed eye-tracking technology to better understand the differences in gaze behavior and decision-making between expert and non-expert basketball referees. The research results confirm that there are significant differences between expert and non-expert basketball referees in terms of the percentage of gaze time in the central area and the outer area, as well as the decision-making accuracy and fixation heat map. However, the study did not find differences among the groups in terms of the number of fixations, the duration of fixations, entropy, and the percentage of fixation time in the invalid area.

Compared with non-expert groups, the decision-making accuracy of expert basketball referees is much better. Expertise has been defined as the ability to consistently demonstrate superior athletic performance (Janelle et al., 2000). Researches have showed that expert officials develop synergistic integration of extensive procedural knowledge (information on “how to” perform a task) and declarative knowledge (understanding the “whats and whys”), enabling them to extract critical information from the environment to anticipate future events (French and Thomas, 1987; McPherson, 1999). This knowledge architecture allows them to anticipate and manage potential “flashpoints” during competitions (Mascarenhas et al., 2005). In other words, the key role of anticipatory ability in the decision-making process (Ericsson and Kintsch, 1995), research has proved that the excellent anticipatory ability of expert referees is inseparable from long-term high-level officiating experience (Schrödter et al., 2023). High-level expected ability mainly stems from the rich practical competition refereeing experience of expert referees or the transfer of sports skills when they are athletes. On the other hand, long-term working memory provides effective support for the decision-making of expert referees (Ericsson and Chase, 1982). When expert referees observe the movements that appear in the video, they will quickly recall from their minds whether the occurrence of the movement is a violation, thereby making decisions promptly (Plessner and Haar, 2006; Ericsson, 2008). Therefore, we believe that different memory retrieval methods will lead to differences in information processing. Because expert referees have rich officiating experience, they will formulate more refined information retrieval strategies and processing methods. When confronted with repetitive scenarios, it can more effectively extract key information from long-term memory, guide visual attention, and make correct decisions. In contrast, non-expert referees rely more on random visual behaviors to collect information when facing complex game scenarios, and they lack the ability of anticipation and long-term working memory. Therefore, expert referees can show higher accuracy of decision making.

There are differences in the performance of expert and non-expert referees in the fixation heat map. From the figure, we can intuitively find that expert referees allocate their attention to the key areas and can effectively suppress the visual information in the outer areas and invalid areas. However, non-expert referees allocate approximately half of the fixation to the outer areas and invalid areas, and the fixation is relatively scattered, making it impossible to quickly and accurately extract key information. Our findings support the results of previous studies. When a high-level basketball referee is refereeing, his attention will be focused on the offensive and defensive players who hold the ball (Klatt et al., 2021). When an attacking player starts with the ball, the expert referee will first observe whether the player will commit a travel violation when they start dribbling. Then officiate the defense (which is also required by the current FIBA refereeing regulations), while visually tracking and searching the defenders for any illegal actions (Ruiz et al., 2024). When the offensive player finishes dribbling and stops, the expert referee will again observe whether the offensive player has committed a travel violation. Then, when the offensive player is about to shoot or pass the ball, the referee will pay attention to whether the defensive player has committed a violation at this time.

In this study, by drawing on the research designs of predecessors, the body parts of athletes were divided into regions of interest (Moore et al., 2019). Our research indicates that the fixations time of expert referees in the central area and the outer area is significantly longer than that of non-expert referees. Previous studies support this result. As an interactor, basketball referees usually need to handle a large number of game clues (MacMahon et al., 2014), and complete the reasonable distribution of attention among the referee’s responsibility area, the ball, players and teammates in the game scene. On the one hand, compared with non-expert referees, expert basketball referees have developed a stable “internal model” for visual search, enabling them to efficiently allocate attention in visual search tasks (Kostrna and Tenenbaum, 2021). In tasks with limited decision-making time, expert referees can better distinguish relevant information sources (the athletic performance of the central area and the outer area) from irrelevant information sources (the athletic performance of the invalid area), and focus their attention on the most important information sources. On the other hand, from the perspective of the information reduction hypothesis, this result indicates that expert basketball referees may learn through experience to optimize the amount of information processed and selectively focus on task-related information (Wu, 2024). We believe that the information reduction hypothesis supports the establishment of an “internal model” for visual search by expert basketball referees. Therefore, expert basketball referees selectively focus their attention on key positions in the central area and the outer area to obtain crucial information and achieve a high decision-making accuracy. However, based on existing knowledge, in the context of fast breaks or transitions between offense and defense, referees also need to monitor the no-ball area. Moreover, the actual refereeing work requires constant adjustment of body position and focus of attention to fully cover the dynamics of the game. This point forms a surface contradiction with our research results. We speculate that the cause of this problem might be the limitations of the laboratory context. In real refereeing, referees compensate for the concentration of gaze through body movements and the switching of gaze points. However, in the video viewing task of this study, the compensation mechanism could not be demonstrated, and expert referees thus showed a more concentrated gaze pattern.

There is no difference in the number of fixations and the fixations duration, which is similar to the results of previous studies. This might be due to the significant differences between the tests completed under laboratory conditions and the positions and perspectives of the referees (MacMahon and Ste-Marie, 2002; Plessner and Betsch, 2001). Previous studies suggested that the number of fixations and the duration of fixations could not be used as important parameters to distinguish experts from non-experts (Catteeuw et al., 2009). However, by further exploring the differences in gaze behavior between expert and non-expert referees through the division of areas of interest, it can be proved that expert referees will spend more time focusing on the important areas and clues of the game (Moore et al., 2019). Expert referees focus their main attention and most of their time on the key information area, while non-expert referees focus their attention and most of their time on the irrelevant information area. Our research also proves this view. In addition, the differences in perceptual and cognitive skills between expert and non-expert referees may also be influenced by other factors. For example, the way video stimuli are presented and the types of stimuli may cause referees to extract different information when watching the materials (Dicks et al., 2010). At present, most studies usually adopt laboratory environments or simulate competitive scenarios. Although they offer strict experimental controls, it is difficult to replicate the dynamics and unpredictability of real competitive conditions. For instance, factors such as crowd noise, high-pressure decision-making and fast-paced interaction. All these may have limited the ecological validity and applicability of the research results. Therefore, future research should incorporate these factors.

This study is the first to explore the difference in entropy between expert and non-expert basketball referees. Although the results show that the fixation entropy of expert basketball referees is lower than that of non-experts. However, no significant difference in fixation entropy was found between experts and non-experts. This is consistent with the results of the study on the gaze behavior of rugby referees (Moore et al., 2019). This might indicate that the accumulated refereeing experience of both expert and non-expert referees enables them to adopt systematic visual search strategies and both strive to focus on more critical information (Haider and Frensch, 1999), it’s just that expert basketball referees pay attention to key information for a longer time. Therefore, this might indicate that a lower fixation entropy predicts more accurate decisions, but research shows that the entropy of sub-elite referees is higher than that of elite referees, and there is no difference in the decision-making accuracy (Moore et al., 2019). Therefore, the interpretation of the result of entropy should be more cautious.

Based on the fact that the expert group and the non-expert group did not show significant differences in the number of fixations and fixations duration and fixation entropy, we speculate that this may be influenced by factors such as the design of the research task or the insufficient sensitivity of the indicators used in the study. Firstly, in terms of task design, the partial absence of real-life situational factors in basketball may instead prevent expert referees from demonstrating their genuine, context-dependent visual search and decision-making advantages. For instance, the game score, the remaining time, the number of fouls committed by the team and the influence of the players, etc. There are the following differences between laboratory conditions and real situations. The richness of perceived information (for instance, in laboratory conditions, referees rely more on foveal vision, while in real matches, referees more often use peripheral vision to monitor the area without the ball); Cognitive and decision-making pressure (for instance, in real competitions, referees might adopt a more conservative gaze pattern due to pressure, or exhibit intuitive visual search strategies based on experience). Task participation and physical participation (for example, in laboratory conditions, physical movement is stripped away, while in a real competition, the referee’s gaze strategy is accomplished through eye-head-body coordination). Secondly, on the selected indicators, it may be impossible to capture the micro-differences in visual information extraction between the expert group and the non-expert group. Some eye movement data failed to form a strong correlation with decision-making accuracy. It is possible that the two groups adopted the same visual search strategy but exhibited different decision-making behaviors, which would result in us not being able to observe significant differences between the groups.

## 5 Conclusion

In conclusion, this study examined the gaze behavior and decision-making accuracy of basketball referees with different experiences when evaluating game scenarios. Compared with non-expert basketball referees, expert basketball referees make more accurate decisions, pay more attention to the central and outer areas, pay less attention to the invalid area, have a smaller entropy, a shorter fixations duration, and a similar number of fixations. This research places greater emphasis on the fact that the gaze behavior of basketball referees is related to the decision-making in the game scene. Therefore, it can be incorporated into the personalized training plan for improving the decision-making ability of basketball referees.

## 6 Limitations and outlook

First, the sample size is relatively small, which is due to the scarcity of the referee group in Chinese’s top basketball league and the extreme difficulty in recruitment (Schnyder et al., 2017). Although the trends and effect magnitudes reported by the research institute have significant reference value and provide a crucial foundation for future large-sample validation studies. However, the post-event statistical test power also fails to reach the standard value, which means that the failure to detect significant differences may result from insufficient statistical test power. Therefore, we encourage researchers to use a larger sample size and more trials in the visual search and decision-making tasks of referees. Second, the video clips were captured by the camera at the position of the lead referee. These video clips help enhance the representativeness of this research task. However, existing studies have shown that different perspectives can affect visual search behavior. Future research should control this by generating unique first-person shots (Spitz et al., 2016). Thirdly, although this study attempts to adopt a more realistic referees’ specific decision-making task than previous studies, this task is still carried out in the laboratory rather than in a natural game context, which limits the representativeness of the task. Since gaze behaviors in different situations may vary due to different task constraints (Dicks et al., 2010), future research should give priority to using mobile gaze tracking devices to enhance the ecological validity of perception-cognitive assessment. Furthermore, eye-tracking technology, when combined with other physiological and cognitive tests (such as EEG) and gesture tracking devices (such as Leap Motion Controller^®^), provides a comprehensive understanding of the factors influencing basketball performance.

## Data Availability

The raw data supporting the conclusions of this article will be made available by the authors, without undue reservation.
